# Gender differences in mental health problems among adolescents and the role of social support: results from the Belgian health interview surveys 2008 and 2013

**DOI:** 10.1186/s12888-018-1591-4

**Published:** 2018-01-10

**Authors:** Filip Van Droogenbroeck, Bram Spruyt, Gil Keppens

**Affiliations:** Sociology Department, Vrije Universiteit Brussel, Pleinlaan 2, 1050 Brussel, Belgium

**Keywords:** Psychological distress, Anxiety, Depression, Gender, trends, social support, adolescent mental health

## Abstract

**Background:**

To investigate how social support relates to mental health problems for Belgian late adolescents and young adults 15–25 years of age. Additionally, we examine changes in mental health problems between 2008 and 2013 and investigate gender differences.

**Methods:**

Multivariate analysis of variance was used to investigate (1) psychological distress, (2) anxiety and (3) depression among 713 boys and 720 girls taken from two successive waves (2008 and 2013) of a representative sample of the Belgian population (Belgian Health Interview survey). Psychological distress was measured by the General Health Questionnaire, anxiety and depression by the Symptom Check-List-90-Revised.

**Results:**

Gender differences were found for psychological distress, anxiety and depression with girls reporting significantly higher scores than boys. Multivariate analysis of variance (MANOVA) revealed that adolescents who are dissatisfied with their social contacts and experience poor social support reported more psychological distress, anxiety and depression. In addition, young adult boys (20–25 years of age) were more likely to experience psychological distress when compared to late adolescent boys (15–19 years of age). Finally, the prevalence of anxiety and depression increased substantially between 2008 and 2013 for girls and to a lesser extent for boys.

**Conclusions:**

Especially girls and young people with poor social support experience mental health problems more frequently than boys and those with strong social support. Improving social support among young people may serve as a protective buffer to mental health problems.

## Background

Mental health has become a priority in public health policy. In 2013 a World Health Assembly resolution was passed by the World Health Organization (WHO) that called for a comprehensive mental health action plan at the national level [1]. In this regard, late adolescents and young adults deserve special attention. Half of all mental health disorders in adulthood start by the age of 14 and three-quarter by the age of 25 [2]. According to the most recent statistics, in any given year 20% of adolescents worldwide experience mental disorders, most commonly anxiety or depression (WHO, 2012). Mental health problems are considered to be some of the most common and yet most stigmatising of conditions [3].

Defining mental health is challenging as it is conceptually ambiguous. In this article we adopt the holistic definition from the World Health Organization [4] which describes mental health as “a state of well-being in which every individual realizes his or her own potential, can cope with the normal stresses of life, can work productively and fruitfully, and is able to make a contribution to her or his community.” Mental health problems in adolescents result from the complex and dynamic interplay between individual attributes and behaviours (e.g., genetic factors, emotional and social intelligence), social and economic circumstances (e.g., experienced social support, poverty, education opportunities), and wider sociocultural environmental factors (e.g., social and economic policies at the national level, discrimination) [5]. These aspects interact with each other in a dynamic way that can either protect, or pose a risk to, mental health. In this article we will investigate how social support and socioeconomic status (SES) are related to three common mental health problems (i.e., psychological distress, anxiety and depression) across gender amongst a representative sample of Belgian young people between 15 and 25 years of age. This age group allows us to compare late adolescents (15–19) with young adults (20–25). Earlier research suggests that the transitional period from adolescence to young adulthood is characterised by changes in people’s psychological attachment styles [6].

Social support can be considered as a form of social capital and is usually defined by structural aspects of people’s relationships (e.g., number of ties, group memberships, etc.) and explicit functions (e.g., emotional, informational, instrumental assistance) they may serve. Social support has been identified as a key protective factor for mental well-being [7–11]. Positive relationships with family and friends are thought to serve as buffers to the negative influences within one’s immediate environment. Although there is no consensus on the mechanisms of how social support positively influences mental health, generally, two broad categories of supportive behaviors are distinguished: emotional sustenance and active coping assistance. Emotional sustenance are demonstrations of caring, valuing, and understanding by (significant) others while active coping assistance relates to supporters giving advice or implementing problem-focused and emotion-focused coping strategies that they would use themselves [7, 12]. Both influence mental health differently, emotional sustenance has primarily an indirect influence through social psychological mechanisms (e.g., social influence/comparison, social control, role-based purpose and meaning, self-esteem, sense of control, belonging) while active coping assistance primarily has a direct influence on mental health (see [12] for an overview of the mechanisms of social support to mental health). Some studies found gender differences for the relationship between social support and mental health problems. Schraedley and colleagues found that the mental health problems of girls were more related to the level of social support than for boys [13]. Furthermore, well-established gender differences exist for mental health problems in general. Depression, anxiety, and psychological distress affect girls to a greater extent than boys across different countries and varying contexts [14, 15]. For these reasons we will pay specific attention to gender differences in the empirical analyses.

We contribute to the existing literature in three ways. First, poor mental health has important consequences for the broader health and long-term development of adolescents and is associated with several health and social outcomes such as unemployment, higher alcohol, tobacco and illicit substances use, adolescent pregnancy, school dropout and delinquent behaviours [16, 17]. In health terms, mental health problems are related to premature mortality (up to 20 years due to suicide or from other diseases that are often left unattended such as HIV, cancers, cardiovascular disease, and diabetes [5, 18]. Suicide is in the top three causes of death for young people between 10 and 25 years of age [19]. The long-term consequences of poor mental health make it important to investigate the determinants of mental health problems at the age when they are known to most likely develop. We adopt a broad focus by studying not only depression but also distress and anxiety for boys and girls separately. Second, to the best of our knowledge this is the first Belgian population-based study on adolescent mental health problems and adds to the scarce population-based research on the relation between social support and mental health problems among adolescents. Third, by using two successive waves of the Belgian Health Survey, we are able to study the evolution in the prevalence of three mental health problems across gender and in this way contribute to the literature that documents evolutions in mental problems [20]. In this regard, several studies indicated an increase in mental health problems over time for girls but not necessarily for boys [20–23]. A recent study by Fink et al. in England indicated that girls but not boys reported more emotional problems in 2014 than in 2009 [20]. More research is needed to assess if these gender-specific trends are also observable in other countries. The period covered by our data (2008–2013) is particularly interesting because it concerns one of the most severe financial and economic crises since WWII with high unemployment rates especially among young people.

## Methods

### Data

The data are based on two successive cross-sectional waves (2008 and 2013) of the Belgian Health Interview Survey (B-HIS) (https://his.wiv-isp.be/). The research protocol was approved by the High Council of Statistics, the ethical committee of the Scientific Institute of Public Health and the Commission for the Protection of Private Life. This ensured that the privacy of the respondents was protected (for more information see https://his.wiv-isp.be/Shared%20Documents/protocol2004.pdf). The B-HIS is held among a representative sample of the Belgian population (15 to 105 years of age) and aims to give an overview of the health status of the Belgian population. It is repeated every 3–5 years and each wave has to result in a net sample of at least 10,000 participants. A multistage sampling design is used, which involves geographical stratification (regions, municipalities), a selection of households within each cluster, and a selection of respondents within each household. The methodology has been described in detail by Demarest and colleagues [24]. Face-to-face interviews with PAPI (paper and pencil interviewing) are used. In addition, self-administered questionnaires were given to the participants older than 15 years, which dealt with more sensitive topics such as mental health problems and alcohol consumption. Instruments were constructed following strict procedures, including mainly standardised instruments from the WHO Consensus on Harmonising Methods and Instruments for Health Surveys, the Dutch Central Bureau of Statistics, the Belgian Centre for Social Policy, and the French National Institute for Health and Medical Research.

### Measures

We focus on three mental health problems: psychological distress, depression and anxiety. These were measured by widely validated scales such as the Symptom Check-List-90-Revised (SCL-90-R) and the General Health Questionnaire (GHQ12). Research has demonstrated that both the SCL-90-R and GHQ12 can be successfully used as a screening instrument to identify mental disorders among young people [25–27].

*Depression and anxiety* were measured by the SCL-90-R, a 90-item instrument which assesses the presence and severity of a wide range of symptoms in an individual in the week prior to the interview [28]. Although the original SCL-90-R instrument comprises nine subscales, several studies failed to identify the nine-factor structure [29–31]. Instead the SCL-90-R shows a better fit as one global index. Still there are good reasons to use the anxiety and depression subscales as separate constructs. First, several studies indicate that the anxiety and depression subscales have been used separately and successfully to screen clients for depression and anxiety [32–35]. Second, previous research has also revealed a frequent temporal sequence where anxiety comes first and subsequently depression [36, 37]. Therefore, using anxiety as a separate construct might facilitate insight into the early identification of depression. In general, anxiety and depression are commonly considered to be comorbid conditions and are correlated [38–40]. Third, we performed confirmatory factor analysis to test a one-factor and two-factor solution. Although both scales have a strong correlation as expected (*r* = .79; *p* < .000) the two-factor model (χ^2^(226) = 1583.3; RMSEA = 0.065 (upper limit 90% 0.068); CFI = 0.910; TLI = 0.900; SRMR = 0.042 – three error-covariances) reveals a better fit than the one factor solution (χ^2^(227) = 1909.2; RMSEA = 0.069 (upper limit 90% 0.075); CFI = 0.889; TLI = 0.876; SRMR = 0.045 – three error-covariances). Based on these three considerations, we report the results for the depression and anxiety subscales separately in this article.^1^

Depression was measured by 13 items (e.g., “Repeated unpleasant thoughts that won’t leave your mind,” α = .89^2^). Anxiety was measured by 10 items (e.g., “Spells of terror or panic,” α = .88^3^). Respondents were asked to rate the intensity of distress by the symptom on a 5-point Likert scale, ranging from 1 “not at all” to 5 “extremely,” with higher scores indicating more complaints. For each indicator, the relevant items were summed and divided by the number of items resulting in sum scales ranging from 1 to 5.

*Psychological Distress* was assessed by the GHQ12 which is designed to detect non-psychotic psychiatric problems [41, 42]. It is a composed index of 12 items (e.g., “Felt constantly under strain?”; α = .83), which are rated by respondents on a 5-point Likert scale ranging from 0 “not at all” to 4 “much more than usual”. The recommended traditional scoring method was used to recode the initial Likert scores in a summed score ranging between 0 and 12 [41].

We included six independent variables. *Age* (0 “15–19 years of age”, 1 “20–25 years of age”) was used as a control variable. We also included *year of the survey* (0 “2008”, 1 “2013”) to assess trends between survey years.

The *highest educational level within the household* (0 “Higher secondary education or lower”, 1 “At least higher education”) and *household income* were considered as a proxy for different levels of SES of the family. The household income variable was divided in quintiles which are based on the income distribution at the Belgian level. Higher scores reflect higher household incomes relative to the Belgian income distribution.

To measure social capital two variables where constructed. The *appreciation of social contacts* (0 “Rather unsatisfied”, 1 “Rather satisfied”) and the *perceived quality of social support* (0 “Poor support”, 1 “Intermediate or strong support”). The correlation between both indicators was .242 (*p* < .001).

### Participants

Currently, five waves of the Belgian Health Interview Survey (B-HIS) are available. For the purpose of our study, only waves which had indicators on social support were used. The response rates for the consecutive waves are: 55% (2008; *N* = 11,254) and 57% (2013; *N* = 10,829). In addition, young people between 15 and 25 years of age (N_total_ = 1433) were selected. Baseline participation for our age range was (*N* = 1976) which implies an item non-response of (*N* = 543). Item non-response analysis indicated that respondents from households with a lower education level were more likely to have missing values for the items concerning health and the other independent variables. This item non-response is closely related to the unit non-response (i.e. failure to participate) as observed in the B-HIS and other national health surveys [24]. In general, the relationship between socially and economically deprived respondents and non-response bias has been well established [43]. Given the positive relationship between being socially and economically deprived and mental health problems the item non-response in the B-HIS might underestimate the prevalence of and social differences in mental health problems and influence our general estimations.

### Analysis

Our analysis is borne out of two parts: first, descriptive statistics are given for all dependent variables. Second, multivariate analysis of variance (MANOVA) was used to assess differences in mental health problems for age, household income, highest educational level in the household, and social support for boys and girls separately. In addition, a dummy variable was constructed to control for the year of the survey and to assess trends. Coefficients with 95% confidence intervals (CIs) were calculated for each predictor. All analyses were carried out in Stata-MP (version 13) [44].

## Results

### Descriptive statistics

Table 1 presents the descriptive statistics for young people in the age group of 15–25 years. Approximately 60% of the investigated young people were between 20 and 25 years, half the sample consisted of girls and about 60% came from a household where the parents’ educational level was not higher than secondary education. The majority of the investigated young people were rather satisfied with the appreciation of their social contacts (94.2%) and perceived an intermediate or strong quality of social support (87%). Anova with post hoc Bonferonni corrections indicated that girls score significantly higher on psychological distress, depression and anxiety than boys. Gender differences for the social economic background and social capital indicators were very small.

**Table 1:** Descriptive statistics (column percentages)

	Male %	Female %	Total %
Year			
2008	60.6	56.1	58.3
2013	39.4	43.9	41.7
Gender			
Male	/	/	49.8
Female	/	/	50.2
Age			
15–19	42.4	38.6	40.5
20–25	57.6	61.4	59.5
Highest educational level within the household			
Secondary education or lower	58.8	58.6	58.7
At least higher education	41.2	41.4	41.3
Appreciation social contact			
Rather unsatisfied	6.2	5.4	5.8
Rather satisfied	93.8	94.6	94.2
Quality of social support			
Poor support	12.3	13.6	13.0
Intermediate/strong support	87.7	86.4	87.0
Reported equivalent household income (Belgian Weighted quintiles – m(S.D.))	2.86 (1.44)	2.81 (1.44)	2.84 (1.44)
Psychological distress^a^ (1–12) M(S.D.)	1.13 (2.11)	1.71 (2.48)	1.42 (2.32)
Depression^a^ (1-5) M(S.D.)	1.27 (0.40)	1.45 (0.57)	1.36 (0.50)
Anxiety^a^ (1-5) M(S.D.)	1.21 (0.37)	1.35 (0.55)	1.28 (0.47)
Total N	713	720	1433

^a^Means between girls and boys on these scales differ significantly, that is, *p <* 0.000 (ANOVA with *post hoc* Bonferroni test)

### Multivariate results

Table 2 shows the results for the selected variables using a multivariate analysis of variance for boys and girls separately. We found similar results for boys and girls. Psychological distress, depression and anxiety was strongly related with our indicators for social capital. In addition, young people who are dissatisfied with their social contacts and experience poor social support were more likely to report psychological distress, anxiety and depression. Although the point estimates of the effect parameters vary, the overlapping confidence intervals indicate that in general the strength of these relations is comparable across the different outcomes. There was no significant relationship for boys or girls between our indicators of social position and the three mental health problems. For other indicators, however, we did find interesting gender differences. Psychological distress was more common among young adult boys (20–25 years old) when compared to late adolescent boys (15–19 years old). No such age differences were found among girls. In addition, the results showed significant and substantial differences in depression and anxiety scores across the two waves between girls and to a lesser extent for boys. The relationship between depression, anxiety and survey year was twice as large for girls when compared to boys. In order to test whether the effect-size for the waves differed between boys and girls, we used the formula proposed by Paternoster et al. to test for the equality of regression coefficients [45]. We found that in a multivariate model the strength of the effect of the year of the survey differed significantly at the *p* < 0.100 level between girls and boys for depression (z = −1.71; *p* = 0.087) and anxiety (z = −1.79; *p* = 0.073). In addition, girls reported more psychological distress, anxiety and depression than boys in a combined multivariate model which controlled for the independent variables (not shown in table).

**Table 2:** MANOVA on psychological distress, depression and anxiety – Unstandardized coefficients and 95% confidence intervals (CI)

	Psychological distress (GHQ 1–12)				Depression subscale (1–5)				Anxiety subscale (1–5)			
	Male		Female		Male		Female		Male		Female	
	b	95% CI	b	95% CI	b	95% CI	b	95% CI	b	95% CI	b	95% CI
Survey year (2013 vs. 2008)	−0.40*	[−0.71;−0.09]	-0.09	[−0.44;0.27]	0.08**	[0.03;0.14]	0.17***	[0.09;0.24]	0.06*	[0.01;0.12]	0.15***	[0.07;0.23]
Highest educational level within the household (At least higher education vs. Higher secondary education or lower)	0.09	[−0.25;0.42]	0.31	[−0.09;0.71]	0.01	[−0.05;0.07]	−0.02	[−0.11;0.07]	−0.02	[−0.07;0.04]	0.01	[−0.08;0.1]
Age (20–25 vs. 15–19)	0.46**	[0.15;0.76]	0.10	[−0.26;0.46]	0.02	[−0.04;0.07]	0.02	[−0.06;0.1]	0.03	[−0.03;0.08]	0.07	[−0.01;0.15]
Reported equivalent household income (Belgian weighted quintiles)	−0.05	[−0.16;0.07]	0.03	[−0.11;0.16]	0.00	[−0.02;0.02]	0.00	[−0.03;0.03]	0.01	[−0.01;0.03]	−0.01	[−0.04;0.02]
Appreciation social contacts (Rather satisfied vs. Rather unsatisfied)	−1.85***	[−2.5;-1.21]	−1.94***	[−2.73;-1.15]	−0.52***	[−0.64;-0.4]	−0.49***	[−0.67;-0.32]	−0.41***	[−0.52;-0.3]	−0.20*	[−0.38;-0.03]
Quality of social support (Poor support vs. Intermediate/strong support)	−0.48	[−0.97;0]	−1.39***	[−1.91;-0.86]	−0.24***	[−0.33;-0.15]	−0.44***	[−0.55;-0.32]	−0.15**	[−0.23;-0.07]	−0.28***	[−0.4;-0.16]
Constant	3.29***	[2.56;4.02]	4.52***	[3.61;5.43]	1.92***	[1.79;2.05]	2.22***	[2.02;2.43]	1.67***	[1.55;1.8]	1.71***	[1.51;1.92]
N	713		720		713		720		713		720	
R^2^ (%)	8.3%		8.4%		18.1%		15.0%		11.9%		6.7%	

* *p* < 0.05, ** *p* < 0.01, *** *p* < 0.001

## Conclusion

Late adolescence and young adulthood are phases in life characterised by profound transitions and changes. The quest for one’s own identity and the search for a way to stand on one’s own feet goes along with feelings of uncertainty and anxiety. While most adolescents are able to successfully cope with these feelings, a considerable group suffers from more serious mental health problems. In this study, we used high-quality representative data from 1433 Belgian late adolescents and young adults gathered in 2008 and 2013. The GHQ12 and SCL-90-R measures showed gender differences in the prevalence of mental health problems which is consistent with previous studies [5, 13, 15, 20, 46]. Boys reported less psychological distress, anxiety and depression than girls. These results are important especially if one takes into account the fact that mental health problems that manifest themselves during late adolescence and young adulthood prove to be good predictors for mental health disorders in adulthood [2]. For this reason health policies should closely monitor the evolutions in the mental health of youth and develop effective prevention strategies. This seems particularly applicable for depression and anxiety among girls for which we found that anxiety and depression increased substantively in comparison to boys in the span of only 5 years’ time. The observed increase in depression and anxiety for girls and to a lesser extent for boys is consistent with previous research in England [20]. On the other hand, boys reported less psychological distress in 2013 when compared to 2008 while for girls we did not find this for psychological distress. These opposite results illustrate the importance of investigating different aspects of mental health and how these are differentially related to gender. The causes of gender differences in mental health problems among adolescents are not fully understood but previous research has indicated that boys may have more difficulties in acknowledging their mental health problems and tend to mask their mental health problems by acting out their difficulties resulting in more externalising disorders that are problematic for others such as antisocial personality disorders and substance abuse or dependence [17]. Girls, on the other hand, report more internalising disorders such as depression and anxiety. These differences between boys and girls may be related to gender conceptions and the socially defined role of women and men which in many societies exposes them to gender-specific stressors. Girls are expected to be more emotionally sensitive [47], suffer more from stressors which involve significant others such as the death of friends or relatives [48], experience more restricted gender roles and body dissatisfaction [49, 50], ruminate more as a coping strategy [51], experience more family violence, abuse and school performance pressure [15, 52], which all have been associated with a greater likelihood of mental health problems. Given that the trend patterns which we found for girls are similar to a recent study in England covering the same time period this might point to the influence of the economic and financial crisis which has been associated with higher levels of mental health problems among adolescents due to increasing youth unemployment and cuts to mental health services [20, 53, 54]. If these societal and economic changes are influencing the mental health of adolescents, the key question raised by our results concerns whether and why these would have a differential impact on boys and girls. Our data does not allow us to answer this question but at least suggest that the impact of societal changes associated with the economic and financial crisis may be much more far-reaching than what is often assumed in the public debates. Biological gender differences and the different societal expectations towards boys and girls which are related to mental health problems call for effective mental health promotion strategies that are adapted to the needs of boys and girls (e.g., see [5, 14] for effective promotion strategies of women’s mental health).

Schraedley and colleagues found that among adolescents the prevalence of mental health problems increase with increasing age [13]. We found that young adult boys (20–25 years old) suffered from more psychological distress when compared to late adolescent boys (15–19 years old). No significant differences were found for girls or for the other mental health problems for boys, but the parameter estimates point towards the same direction. A plausible explanation for these findings is that the border between late adolescence and young adult almost coincides with the end of compulsory education in Belgium (18 years of age). People who leave compulsory education either go to higher tertiary education or enter the labour market. In both cases young people are confronted with many new experiences, expectations, potential unemployment, and responsibilities which may result in increased stress and along this way affect mental health. Although our data does not allow a strict empirical test of this idea, several other studies provide empirical support for the latter reasoning [53, 55]. These findings illustrate the importance to investigate these mental health problems separately and not combine them into a general construct.

In stressful situations it is good to have people to rely on. Indeed, one of the main contributions of this study is that it shows the crucial importance of a rewarding social network regarding young people’s mental health problems. Late adolescents and young adults who are satisfied with their social contacts and/or feel strongly supported by others, report a less anxiety, depression, and psychological distress. In this context it is important to stress that social relationships do not only provide support in case of problems (curative function) but also opportunities for (different kinds of) voluntary action. Doing something meaningful for others and/or society is an effective means to search for one’s own identity and is associated with greater (mental) health (prevention function) [56]. This is important because mental health problems often prove to be difficult to cure.

### Limitations and further research

Our results raise three questions which cannot be answered with the data we have at our disposal but provide an excellent starting point for further research. *First,* as our results are based on correlational data caution is warranted when interpreting our results. This implies that there could be reversed causality. Social support can influence mental health but existing mental health problems are also likely to affect the number of social ties and type of support an individual receives. Developing effective prevention strategies require that we get a better grip on the causal mechanism behind the observed differences and evolutions. Our results suggest that especially the transition from compulsory education to tertiary education/entering the labour market seems to be a particularly relevant life change to study with a longitudinal design that allows the assessment of differences within individuals over time.

*Second,* the importance of having a rewarding social network for young people’s mental health raises questions concerning which social relationships are more important than others, how different social relationships interact, and what aspects of social relationships are beneficial for mental health. Indeed, late adolescents and young adults have relationships with many other people. Especially the relative importance between adults (i.e. parents, family, teachers…) and peers seems worthy to investigate [10]. To answer these questions network data provide much more opportunities when compared to the common samples of individuals as used in classical health surveys. In addition, the relationship between the different components of social support and mental health problems can be disentangled. Research from Van Voorhees suggests that for young people’s mental health the feeling to be accepted among peers rather than warmth or support is a critical protective factor in the peer context. For the relationship with the parents on the other hand, a sense of closeness and warm relations play an important protective role [46].

Third, quite surprisingly no important net differences were found according to indicators that reflect the social position (i.e., highest educational level in the household and household income). One possible explanation for this is the selection bias and higher non-response among socially and economically deprived respondents. This potentially flattens differences between social strata and underestimates the general prevalence of mental health problems because research has indicated that people from socially disadvantaged backgrounds are more likely to report worse (mental) health. This is a problem most (national health) surveys are confronted with. The B-HIS takes measures both in the design and management of the survey to reduce the impact of unit non-response on the representativeness of the survey results but still this selection bias potentially affects our results and should be taken into account when interpreting our findings. A second explanation could be that the measures we relied on mainly refer to the social *context* in which late adolescents and young adults live. While young people who live in socially and economically deprived families can be expected to have to cope with more (severe) problems, the extent to which they successfully manage to do so may be stronger related to their *own* social position. Indeed, one of the distinguishing features of late adolescence and young adulthood is that people find themselves at the crossroad of the ascribed (family) status and the achieved personal status. Although sociological research continues to show persistent social reproduction, the intergenerational transmission of social position is far from perfect [57]. Further research should deepen our understanding of the impact of social mobility on young people’s mental health [58]. Another reason why social differences in mental health problems may be rather modest among young people is that mental health is the outcome of the balance between personal experiences *and* expectations. It is known that parents in middle and higher class families often have very high expectations regarding their children’ educational and labour market success [59]. This renders it plausible that the absence of a uniform relationship between indicators for social position and mental health problems may conceal that in different social environments poor mental health is caused by different factors (e.g., material deprivation in lower classes; unrealistically high expectations among the middle and higher classes). At this point only research that includes more fine measures of expectations are able to enhance our understanding of the relationship between social position and mental health problems.

## Footnotes

1 Results for a composed depression/anxiety indicator will be made available upon simple request.

2 Fit indices from confirmatory factor analysis: (χ^2^ (65) = 535.664, RMSEA = 0.071 CFI = 0.935, TLI = 0.922, SRMR = 0.036).

3 Fit indices from confirmatory factor analysis: (χ^2^ (62) = 328.207, RMSEA = 0.055, CFI = 0.963, TLI = 0.954, SRMR = 0.028).
